# Pupal Age Estimation of *Sarcophaga peregrina* (Diptera: Sarcophagidae) at Different Constant Temperatures Utilizing ATR-FTIR Spectroscopy and Cuticular Hydrocarbons

**DOI:** 10.3390/insects14020143

**Published:** 2023-01-31

**Authors:** Yanjie Shang, Yakai Feng, Lipin Ren, Xiangyan Zhang, Fengqin Yang, Changquan Zhang, Yadong Guo

**Affiliations:** 1 Department of Forensic Science, School of Basic Medical Sciences, Central South University, Changsha 410013, China; 2 Department of Forensic Medicine, School of Basic Medical Sciences, Xinjiang Medical University, Urumqi 830017, China

**Keywords:** forensic entomology, age estimations of pupae, ATR-FTIR, cuticular hydrocarbons, *Sarcophaga peregrina*

## Abstract

**Simple Summary:**

The precise pupal age estimates of *S. peregrina* are significant for estimating the minimum postmortem interval (PMImin) in forensic investigations. In the present study, we first investigated the potential of attenuated total reflectance Fourier transform infrared (ATR-FTIR) spectroscopy and cuticular hydrocarbons (CHCs) for the age estimations of *S. peregrina* pupae at different constant temperatures (20 °C, 25 °C, and 30 °C). The orthogonal projections to latent structure discrimination analysis (OPLS-DA) and partial least squares (PLS) models were used to distinguish the pupae samples of different developmental ages.

**Abstract:**

*Sarcophaga peregrina* (Robineau-Desvoidy, 1830) (Diptera: Sarcophagidae) is a forensically important flesh fly that has potential value in estimating the PMImin. The precise pupal age estimation has great implications for PMImin estimation. During larval development, the age determination is straightforward by the morphological changes and variation of length and weight, however, the pupal age estimation is more difficult due to anatomical and morphological changes not being visible. Thus, it is necessary to find new techniques and methods that can be implemented by standard experiments for accurate pupal age estimation. In this study, we first investigated the potential of attenuated total reflectance Fourier transform infrared (ATR-FTIR) spectroscopy and cuticular hydrocarbons (CHCs) for the age estimations of *S. peregrina* pupae at different constant temperatures (20 °C, 25 °C, and 30 °C). The orthogonal projections latent structure discrimination analysis (OPLS-DA) classification model was used to distinguish the pupae samples of different developmental ages. Then, a multivariate statistical regression model, partial least squares (PLS), was established with the spectroscopic and hydrocarbon data for pupal age estimations. We identified 37 CHCs with a carbon chain length between 11 and 35 in the pupae of *S. peregrina*. The results of the OPLS-DA model show a significant separation between different developmental ages of pupae (R2X > 0.928, R2Y > 0.899, Q2 > 0.863). The PLS model had a satisfactory prediction with a good fit between the actual and predicted ages of the pupae (R2 > 0.927, RMSECV < 1.268). The results demonstrate that the variation tendencies of spectroscopy and hydrocarbons were time-dependent, and ATR-FTIR and CHCs may be optimal for the age estimations of pupae of forensically important flies with implications for PMImin estimation in forensic practice.

## 1. Introduction

Estimating the minimum postmortem interval (PMImin) is a crucial link in the examination of criminal cases, and entomological evidence provides a new perspective and direction [1]. Necrophagous flies are the most common entomological evidence on corpses and are often used to estimate the PMImin based on their development stage and age on corpses [2]. Therefore, the precise determination age of necrophagous flies is vital for estimating the PMImin in forensic investigations [3].

The pupal period is an important development stage of necrophagous flies and could last half of the total time of immature development, which means this period has great significance in PMImin estimation [4]. However, due to the lack of anatomical and morphological obvious changes, it is increasingly difficult to estimate aging during this period [5]. Many methods and technologies have been applied to estimate the age during the pupal stage of flies, including puparium color variation [6], intrapuparial morphological characteristics [7], differentially expressed genes (DEGs) analysis [8], micro-computed tomography (µ-CT) imaging [9], and hyperspectral imaging (HSI) [10]. Although these methods have provided some benefits for estimating the age of the pupal stage of flies, it is still necessary to explore some new methods that are quick, easy to operate, and can be implemented by standard experiments for accurate pupal age estimation of forensically important flies.

Vibrational spectroscopy, attenuated total reflectance-Fourier transform infrared (ATR-FTIR) spectroscopy, is a potent analytical method frequently used to detect changes in the bonding type, functional groups, and molecular conformations of biochemical composition, potentially providing the biochemical fingerprint of biological samples under investigation [11]. ATR-FTIR spectroscopy analysis has the characteristics of being quick, low-cost, and user-friendly, providing more spectral information variations to distinguish different samples [12]. A portable and handheld FTIR instrument makes the technique very adaptable for use in forensics [13]. Currently, ATR-FTIR spectroscopy is widely used in biological evidence analysis and forensic science, including the species identification of semen stains [14], blood [15], hair [16], and traumatic brain injury identification [17]. However, there have been few reports on the application of FTIR in forensic entomology [18], especially in the age estimation of flesh flies.

The main components of insect cuticles are cuticular hydrocarbons (CHCs), which play important roles in the formation of insect cuticles, the growth development of insects, limitation of water loss, and the promotion of intraspecies information exchange, mainly consisting of n-alkanes and branched alkanes and alkenes [19]. The identification of CHC profiles by gas chromatography-mass spectrometry (GC-MS) has been proven as a potential method in forensic entomology, including the species identification of necrophagous flies [20], adult fly age estimations in forensically important calliphoridae species [21], predicting the weathering time of puparia [22], and distinguishing the geographic locations of empty puparia [23]. They all provide a reference for the CHC study of necrophagous flies. However, the applications of chemical profiles in the age estimation of forensically relevant sarcophagidae are still limited.

The *Sarcophaga peregrina* (Robineau-Desvoidy, 1,830) (Diptera: Sarcophagidae) is a significant species in ecological, medical, veterinary, and forensic entomology contexts and it is also associated with human habitations and can be found in human cadavers [24]. *S. peregrina* is a typical holometabolous insect that must go through larval–pupal metamorphosis to molt into adults, with the pupal stage lasting up to 50% of the whole growth phase and even several weeks at low temperatures [25]. Thus, the precise age estimation of pupae is important in forensic entomology for the application of *S. peregrina*. Pupae metamorphosis is a complex developmental process that involves significant biochemical composition changes, such as hydrocarbons, proteins, carbohydrates, DNA, and RNA [26].

In the present study, *S. peregrina* adults were captured from a pig carcass, and a laboratory population was established. The spectroscopy characteristics and CHCs profiles during the pupal developmental stages of *S. peregrina* at different constant temperatures (20 °C, 25 °C, and 30 °C) were investigated and analyzed by ATR-FTIR and GC-MS, and their potential value in pupal age estimation was explored. This study provides important methods of age estimation of the pupal stage of *S. peregrina*, and ATR-FTIR and CHCs may be utilized for the age estimation of forensically important flies to help determine more precise PMImin values in forensic science.

## 2. Materials and Methods

### 2.1. Insect Rearing and Sample Collection

In July 2017, *S. peregrina* was obtained from Guo’s Lab (Hunan, China), which was captured from a pig carcass in Changsha city in China (28°12′N, 112°58′E). Species identification was performed using the traditional method according to its morphological characteristics [27] and the molecular identification method [5] before the establishment of the lab colony. Adults of *S. peregrina* were placed in an insect cage (35 × 35 × 35 cm^3^) with natural illumination and temperature, with a humidity of 75 percent. Milk powder was mixed with sugar at a ratio of 1:1, and fresh water was provided. The colonies were kept for five generations, with the number of adults between 1000 and 3000, before starting this study.

To induce larviposition, 20 g of fresh pig lung was placed in a 10 cm culture dish and in rearing cages. The resulting larvae (ca. 1500) produced within 1 h were separated into three groups each (ca. 500 larvae) and placed in three rearing boxes (17 by 12 by 8 cm) with sawdust at the bottom. A climate box (GS110-3, Guansen Biotechnology Co., LTD, Shanghai, China) was used to keep the rearing boxes at constant temperatures of 20 °C, 25 °C, and 30 °C with 75% humidity and a photoperiod of 12:12 (L/D). Fresh pig lung was supplied regularly ad libitum to meet the nutritional needs of the larvae until pupation.

The first sampling began when ca. 50% of the post-feeding larvae formed white puparia. A total of 20 pupae were sampled every 24 h until adult eclosion. Samples from each collection were divided equally into two for ATR-FTIR and CHCs analysis. A 5 mL cryovial was used to collect samples and store them at −80 °C until analysis. The experiments were repeated three times in different incubators at temperatures of 20 °C, 25 °C, and 30 °C. Pupal development lasted 16 days at 20 °C, 10 days at 25 °C, and 8 days at 30 °C. There were 960 pupae collected at 20 °C, 600 pupae at 25 °C, and 480 pupae at 30 °C.

### 2.2. Attenuated Total Reflectance–Fourier Transform Infrared (ATR-FTIR) Analysis

We cleaned the pupal tissue using ultrapure water and blotted it dry with filter paper. One whole pupae tissue was ground in a mortar with liquid nitrogen. Liquid nitrogen continued to be added during the grinding, and then the ground pupal tissue was transferred into 1.5 mL EP tubes and prepared for inspection. Three biological replicates were performed. All spectra were obtained by an 850 FTIR spectrometer equipped with a ZnSe crystal ATR accessory (Tianjin Guangdong Technology Development Co., Ltd., China). The detection window of the ATR accessory was cleaned with distilled water or anhydrous ethanol before each measurement, and the blank background spectra were collected before sampling [28]. To collect the spectra, homogenized tissue from the sample was placed on the ATR accessory. A total of three spectra were collected at a resolution of 4 cm^−1^ in the frequency range of 4000–900 cm^−1^, with 32 scans for each pupal sample. A total of nine spectra were obtained for each pupal development age (days) of *S. peregrina* at each constant temperature (20 °C, 25 °C, and 30 °C) and averaged to a single spectrum to reduce systematic errors. The sample spectra were automatically removed from the background spectra that were gathered on the empty ATR window.

The OMNIC software package (version 9.2) (Thermo Fisher Scientific, Bothell, WA, USA) was used to analyze the spectral data. Unwanted background information was reduced via spectral pre-processing. To lessen the impacts of the size and light scattering of the spectra that occurred due to the sample thickness, the standard normal variate (SNV) was used for all spectra of the sample [29]. In addition, all spectral data were pre-processed by smoothing denoising (Savitzky-Golay convolution algorithm, smoothing points 15). Ultimately, the spectral band between 1800 and 900 cm^−1^, also known as the “biological fingerprint” region, was applied to the following analysis [30].

A supervised statistical method of discriminant analysis was performed using orthogonal partial least squares discriminant analysis (OPLS-DA) [31]. Multivariate regression using partial least squares (PLS) is a common statistical technique [32]. After the data filtering, the OPLS-DA modeling was applied to visualize the variation in the ATR-FTIR spectroscopy of different pupae ages (days) of *S. peregrina* at different constant temperatures (20 °C, 25 °C, and 30 °C). In addition, a PLS regression model for the pupal age estimations was established with the spectroscopy data. The interpretability and reliability of the model were verified by a permutation test. The OPLS-DA and PLS modeling was conducted in SIMCA 14.1 software. The contribution of the factors to the model was evaluated by the variable importance of projection (VIP) value, the more significant a variable was to the model, the higher the VIP value for that variable would be [31]. In accordance with the OPLS-DA and PLS models, we selected spectroscopy with a VIP value > 1.0. The variation trend of the absorbance of these spectroscopies at various pupal development ages was then produced and exhibited in OriginPro software (version 8.6).

### 2.3. Cuticular Hydrocarbon (CHC) Analysis

We cleaned the pupal tissue using ultrapure water and blotted it dry with filter paper. Then, one whole pupae tissue at each pupal development age (days) of *S. peregrina* at each constant temperature (20 °C, 25 °C, and 30 °C) was placed in a 2 mL glass vial with 1 mL of hexane and left at room temperature for 1 h [31]. The liquid was then transferred using a syringe filter with a nylon membrane with a 0.45 m aperture. The liquid was then vacuum-dried and redissolved in 200 ul hexane for the following GC-MS analysis. Four biological replicates were performed.

The CHC analysis was conducted using GC-MS (Agilent Technologies, 7890B-5977A GC/MSD) equipped with a DB-5MS capillary column (30 m × 0.25 mm × 0.25 μm). At 250 °C, 1 μL of liquid was splitless injected. The oven temperature schedule started at 50 °C for 2 min, then rose to 200 °C at 25 °C/min, 260 °C at 6 °C/min, 300 °C at 3 °C/min, and then held for 15 min. The temperature of the interface was 280 °C. Ultrapure helium was employed as the carrier gas. The ion source temperature was 230 °C, and the electron impact mode was 70 eV. An external standard was employed so that the mixture of n-alkanes from C7-C40 dissolved in 1 mL of hexane. We used the software of MSD ChemStation Data Analysis F.01.03 to draw the peak areas and only included the compounds with percentages of peak areas above 0.5%. We used a library search (NIST14), Kovats Index [33], and the literature [34] to identify the hydrocarbons. Unknown compounds and those with less than 0.5% are not presented in the tables.

The OPLS-DA and PLS models were applied to present the CHC data filtered for different pupae ages (days) of *S. peregrina* at different constant temperatures (20 °C, 25 °C, and 30 °C). The interpretability and reliability of the model were verified by a permutation test. The OPLS-DA and PLS modeling was conducted in SIMCA 14.1 software. The VIP value (VIP > 1.0) was used to track how much each variable contributed to the models. The hydrocarbons were selected according to the VIP parameter > 1.0 of the pupal age of *S. peregrina* at different constant temperatures from the OPLS-DA and PLS models (Table S2). In addition, the simulation equations and coefficient of determination (R2) were used to show the relationship between the percentage of the hydrocarbons of VIP parameter >1 (*y*) and the pupal age (*x*) of S. peregrina at 20 °C, 25 °C, and 30 °C. OriginPro version 8.6 was used to construct and display the fitting equation.

## 3. Results

**Figure 1:**
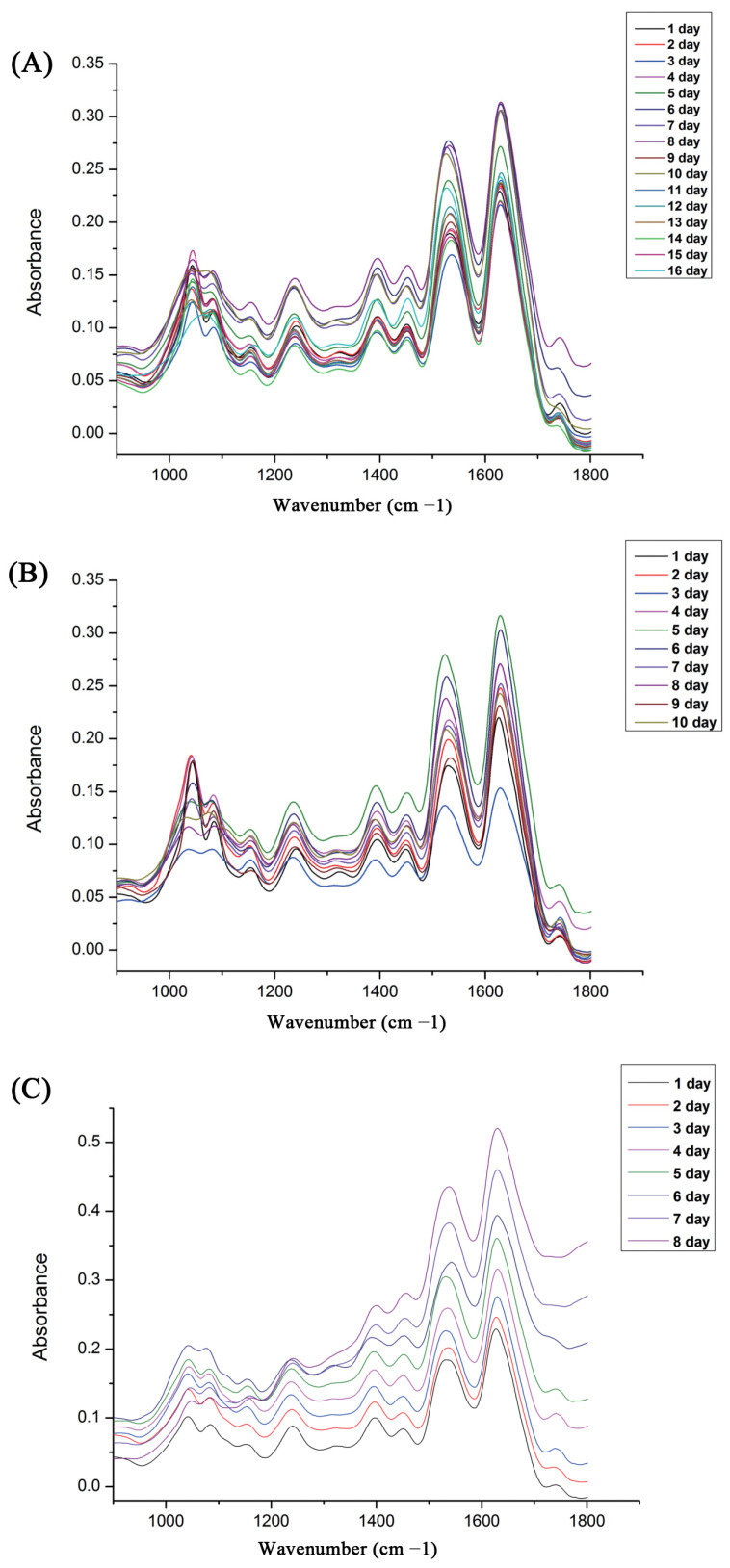
The average ATR-FTIR spectra obtained from different pupae age (days) of *S. peregrina* at different constant temperatures (**A**) 20 °C, (**B**) 25 °C, (**C**) 30 °C.

For the ATR-FTIR analysis, Figure 1 shows the comparison of the average spectra of each pupal development age (days) of *S. peregrina* at each constant temperature (20 °C, 25 °C, and 30 °C) in the range of 1800–900 cm^−1^, and the absorption bands were mainly located in the proteins, nucleic acids, carbohydrates, and lipids. With reference to a previous study [11], the major FTIR spectrum identifications are shown in Table 1. Two prominent peaks in the range of 1700 to 1500 cm^−1^ were connected with proteins, including amide I and amide II in the bands at about 1647 cm^−1^ and 1540 cm^−1^ [32]. Major peaks in the spectrum region of 1100–1000 cm^−1^ were connected with the PO2-symmetric stretching and C-O(H) vibrations, which are prevalent in DNA, RNA, phosphoric acids, carbohydrates, and other biological molecules [28]. In addition, it was found that the mean spectra of pupae at various developmental stages showed distinct variations, which indicates that the spectral characteristic could have the ability for the age estimation of pupae of *S. peregrina*.

For the CHC examination, the CHC profiles of *S. peregrina* pupae at different constant temperatures (20 °C, 25 °C, and 30 °C) showed that a total of 37 hydrocarbons were recognized by GC-MS examination in the pupae, and 13 n-alkanes, 20 branched alkanes, and 4 alkenes were included with a carbon chain length between C11 and C35 (Table S1). We found that the compound classes of hydrocarbons increased with the increase in temperature from 20 °C to 30 °C, and the percentage composition of long-chain hydrocarbons, such as C24, decreased regularly with the pupal developmental age (days) of *S. peregrina*.

### 3.1. OPLS-DA Model Analysis

**Figure 2:**
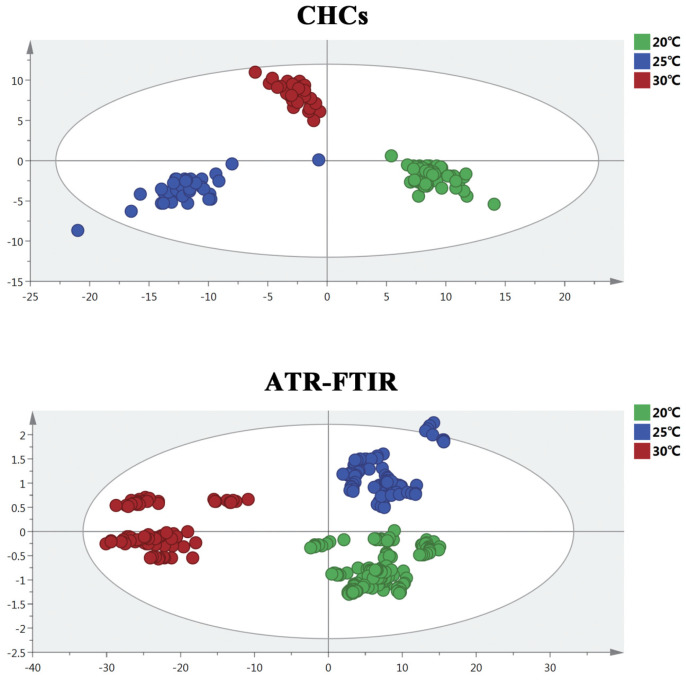
The OPLS-DA model showed clearer visual separation between different constant temperatures based on CHC profiles and ATR-FTIR spectroscopy of *S. peregrina*.

**Figure 3:**
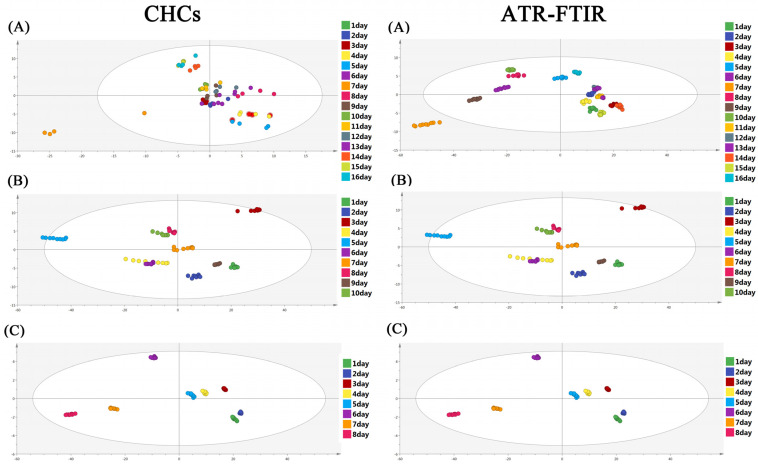
The variations in the CHC profiles and ATR-FTIR spectroscopy of different pupae ages (days) of *S. peregrina* at constant temperatures (**A**) 20 °C, (**B**) 25 °C, (**C**) 30 °C were visualized by OPLS-DA modeling.

The OPLS-DA model was applied to present the differences in the ATR-FTIR spectra and the CHC distribution of *S. peregrina* at different constant temperatures (20 °C, 25 °C, and 30 °C), as shown in Figure 2. The results show that there were obvious differences in the hydrocarbons and spectra of the pupae at different temperatures. To ascertain the differences in the ATR-FTIR spectra and the CHC distribution of *S. peregrina* at different developmental ages of pupae at different temperatures, OPLS-DA modeling was conducted again, as shown in Figure 3. The spectra and hydrocarbons of pupae at similar developmental ages were clustered well. The evaluation results of the OPLS-DA model show that it had reasonable interpretability at different developmental ages of the pupae at different temperatures (R2X > 0.928, R2Y > 0.946, Q2 > 0.863 in CHCs; R2X = 1, R2Y > 0.899, Q2 > 0.953 in ATR-FTIR) (Table 2) and indicated significant differences between the spectra or hydrocarbons of different developmental ages of the pupae. Moreover, a permutation test was performed, and the intercept of Q2 was less than 0, indicating that the model was acceptable (Table 2, Figure S1).

### 3.2. PLS Model Analysis

**Figure 4:**
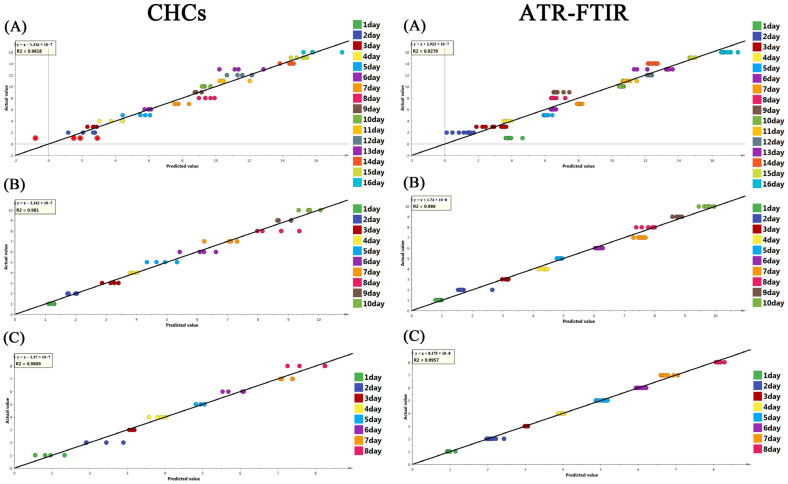
The cross-validation results of the PLS regression model showing the predicted value and the actual value of the pupae age (days) of *S. peregrina* based on CHC profiles and ATR-FTIR spectroscopy at constant temperatures (**A**) 20 °C, (B) 25 °C, (**C**) 30 °C. The black line represents the reference line, where, when the predicted scores are closer to it, the higher the goodness of fit is.

Further examination was performed to investigate whether these spectral or hydrocarbon changes could be utilized for evaluating the pupal age of *S. peregrina* at different constant temperatures (20 °C, 25 °C, and 30 °C). The chemometrics (PLS model) were constructed based on the ATR-FTIR spectra or the CHC dataset, and the detailed results are shown in Table 3. Satisfactory performance was found for the PLS model, with a high R2 (R2 > 0.9618 in CHCs; R2 > 0.927 in ATR-FTIR spectra) and a low error margin for RMSECV (RMSECV < 1.067 in CHCs; RMSECV < 1.268 in ATR-FTIR spectra) in the cross-validation. The results show that the most scattered points of each group are located near the reference line in Figure 4. In addition, the results of the permutation test indicate that the model was acceptable in predicting the pupal age in independent samples (Figure S2). The results show that the ATR-FTIR spectra or CHC characteristics have the potential to be used to predict the pupal age of *S. peregrina*.

### 3.3. Variation Tendency with VIP Parameter > 1.0

**Figure 5:**
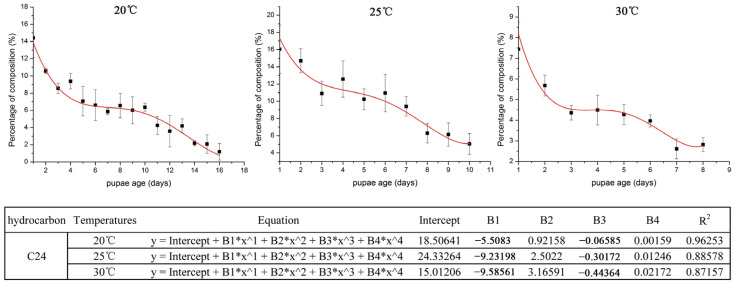
The variation tendency of C24 with a VIP parameter > 1 at different constant temperatures (20 °C, 25 °C, and 30 °C). The *Y*-axis of the tendency chart represents the percentage of the related composition, while the *X*-axis shows the development time of the pupae. The table under the figure shows the parameters of the equation.

The hydrocarbons were selected according to a VIP parameter > 1.0 from the OPLS-DA and PLS models (Table S2). We found that the C24 was present in all three different temperatures (20 °C, 25 °C, and 30 °C) and was shown to be significantly time-dependent and steadily decreased in the different pupal developmental stages, which could be used for age estimations of pupae of *S. peregrina* (Figure 5).

**Figure 6:**
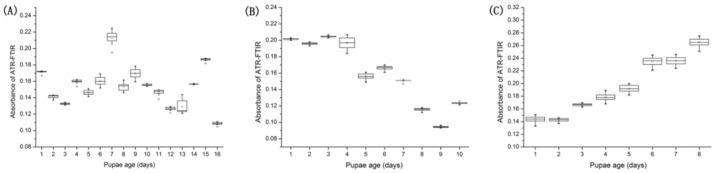
Absorbances of the ATR-FTIR spectral wavenumbers at 1039.45 cm^−1^, 1041.38 cm^−1^, 1043.31 cm^−1^, 1045.24 cm^−1^, 1047.17 cm^−1^, 1049.09 cm^−1^, and 1051.02 cm^−1^ were plotted against the pupae age (days) of *S. peregrina* at different constant temperatures of (**A**) 20 °C, (**B**) 25 °C, and (**C**) 30 °C and are displayed using boxplots. The solid lines within the boxplots represent the median of each pupae development time, the 75% and 25% quartiles are represented by the upper and lower borders of the box, and the 97.5% and 2.5% quantiles are represented by the upper and lower whiskers of the box, respectively. Outliers are represented as asterisks.

The FTIR spectra were also selected from the OPLS-DA and PLS models according to the VIP value (Table S3). We found that these spectra’s range of 1100–1000 cm^−1^ (VIP value > 1.0) mainly involved those bands assigned to proteins, carbohydrates, DNA, and RNA, and played an important role in estimating the pupal age of *S. peregrina* (Figure 6).

## 4. Discussion

The area of forensic entomology has become an increasingly important aspect of forensic science, especially when it comes to PMImin estimation [35]. The pupal stage can account for up to 50% of the developmental time, which means that they will be commonly found, and this stage has great significance in PMImin estimation [36]. Shang et al. reported a case in which light brown to dark brown pupae were found on the corpse and the death scene. This indicates that the accurate determination of the pupal age is a crucial task for using necrophagous flies to estimate PMImin [37]. Traditional methods, such as length and weight measurements used to determine the age of larvae, cannot be applied because the length of a pupa is stable, and the weight of a pupa is a very variable factor [38]. Therefore, it is very important to find more advanced methods for quickly and accurately estimating the pupal age and then estimating PMImin.

Insect metamorphosis is a complex process that includes a series of specific developmental events and programmed cell death to remodel the insect architecture through cell proliferation and differentiation, and many biochemical composition changes occur during this process [39]. ATR-FTIR may be used to evaluate the variations in biochemical composition in insect metamorphosis development based on the absorbance properties of spectroscopy since many functional groups within the biochemical composition have corresponding absorption peaks in the FTIR spectrum [40]. FTIR spectroscopy techniques have been used in some entomological fields, such as the taxonomic discrimination of species [41], larval age prediction [42], toxic substances identification and discrimination in necrophagous flies in entomotoxicology [43], and the detection of the infestations of insects in stored products. Likewise, Pickering et al. used ART-FTIR spectroscopy to correctly classify the life-cycle stages of insects [18]. In our study, we first investigated ATR-FTIR spectroscopy variation during the different pupal developmental stages of *S. peregrina* at constant temperatures (20 °C, 25 °C, and 30 °C). We found that the spectra had significant differences in the developmental stage of pupae, mainly concentrated from 1700 to 1500 cm^−1^ and from 1100 to 1000 cm^−1^, and these spectra mainly involved the bands assigned to proteins, carbohydrates, DNA, and RNA, which is consistent with studies on molecular biology [44]. During the pupal developmental process, the biochemical composition, including nucleic acids, proteins, lipids, and carbohydrates, undergoes drastic changes and regularly changes throughout maturation, which is important for age estimation [45]. Both the classification model and regression model showed that ATR-FTIR combined with the chemometrics could be used to estimate the age of the *S. peregrina* pupae at different temperatures.

Insect mobility, mechanical support, body form, and normal growth are all maintained by the cuticle, which is the outermost layer of the insect body [46]. The cuticle periodically degrades and rebuilds and is important for growth during the molting and metamorphosis of insects [47]. During insect metamorphosis, cuticle degradation undergoes two distinct but causally related processes. Firstly, the larval cuticle degrades to form the pupal cuticle. Secondly, the pupal cuticle degrades to form the adult cuticle [48]. This provides the theoretical basis for the estimation of the developmental age of insects by their cuticular hydrocarbons. Studies on the CHCs of insects for age estimation have already been reported, including the predicted larval age of *Chrysomya albiceps* [49], *Chrysomya rufifacies* [50], the age estimations of the adult fly [21], and empty puparia [51] of *Lucilia sericata* and *Calliphora vicina*. Our study tried to investigate the CHC profiles of different pupal developmental stages of *S. peregrina*, and 37 hydrocarbons were identified in pupae by GC-MS. The high-molecular-weight hydrocarbons of C20–C40 were determined to be the most significant chemicals in the insect cuticle in certain investigations [52], and similar results were found in our study, in which the CHCs with carbon chain lengths from C11 to C35 were identified in *S. peregrina*.

Furthermore, the temperature has a significant effect on the variation in hydrocarbons in insects, and the types and contents of hydrocarbons in insects vary significantly under different temperatures [22]. Zhang et al. reported the CHC composition of the life cycle of *S. peregrina* at a 25 °C temperature, but there was only one temperature involved, and samples were taken every 48 h at the pupal stage [34]. A wider temperature range and more detailed time intervals of sampling are needed for the practical application of CHCs in forensic entomology, especially the age estimation of flies. In this study, we investigated the CHC profile variation during the pupal developmental stages every 24 h of *S. peregrina* at three temperatures (20 °C, 25 °C, and 30 °C), representing the common survival temperature in the pupal stage of *S. peregrina* [25]. We found that the compound classes of hydrocarbons increased with the increases in temperature from 20 °C to 30 °C, and the percentage composition of long-chain hydrocarbons, such as C24, decreased regularly with the pupal development of *S. peregrina*. It might be that higher temperatures increased biodegradation and accelerated the weathering of the surface pupal cuticle, leading to an increase in the CHCs in pupae [31]. In addition, we found that the separation between the age classes from CHC analysis in Figure 3 is better with increasing temperatures from 20 °C to 30 °C and shows a clear separation at 30 °C. It might be that between 20 °C and 30 °C, with increasing temperature, development time decreased (pupal development lasted 16 days at 20 °C, 10 days at 25 °C, and 8 days at 30 °C), but the process of metamorphosis development and periodical variation of the pupal cuticle of *S. peregrina* is the same, which could lead the CHCs to change faster and more differently in everyday high temperatures [26].

The study has shown that heavier long-chain hydrocarbons play an important role in the stability of the cuticle [19]. The cuticle is periodically degraded and rebuilt, which is important for growth during the molting and metamorphosis of insects. During insect metamorphosis, cuticle degradation undergoes two distinct but causally related processes. First, the larval cuticle degrades to form the pupal cuticle, and second, the pupal cuticle degrades to form the adult cuticle [53], and the pupal cuticle periodically degrading could lead to the percentage composition of longer carbon chain CHCs decreasing regularly in the pupal stage. Results of both models in this study show significant differences in the CHC profiles at different developmental stages and have the potential to estimate the age of pupae of *S. peregrina*.

However, before our results can be translated into a forensic technique for estimating the PMI, there are still substantive uncertainties that need to be considered, due to the data of ATR-FTIR and CHCs in our study only coming from constant laboratory conditions. Most studies, including ours, were focused on the constant temperature, due to their controllability in the laboratory, but compared with constant temperatures, the fluctuating temperatures represent a more realistic scenario that occurs in the field [54]. These studies of *diaphorencyrtus aligarhensis* [55] and *tamarixia radiata* [56] provide strong evidence that daily temperature fluctuations significantly affected the development times (and longevity) of parasitoids studied, resulting in marked deviations and potentially erroneous predictions when compared to their constant temperature regimen counterparts. In addition, studies discovered that the effects of constant and fluctuating temperatures on the development of flies, such as *Aldrichina grahami* [54], *Calliphora vicina,* and *Calliphora vomitoria* [57], showed that fluctuating temperatures slowed development, but studies of *Sarcophaga argyrostoma* and *Lucilia illustris* [57] found that development was accelerated. Studies have found that the change in development time at fluctuating temperatures compared with constant temperatures will lead to inaccurate age estimation and further lead to inaccurate PMImin estimation [57]. Thus, when considering cases in fluctuating temperatures, our models may give a greater error for PMI estimation. Studies across a broader set of fluctuating temperature regimes on ATR-FTIR spectroscopy and CHCs of flesh flies are, therefore, encouraged in the future, so that more realistic effects of temperature on the biological parameters of flesh flies can be understood.

## 5. Conclusions

The precise pupal age estimation of *S. peregrina* is important for PMImin estimation in forensic investigations. In this study, the variation tendency of ATR-FTIR spectroscopy and CHCs during the different pupal developmental stages of *S. peregrina* at the different temperatures of 20 °C, 25 °C, and 30 °C were investigated and analyzed. Both the classification model and regression model showed that ATR-FTIR and CHCs, combined with chemometrics analysis, had the potential to be used to more precisely estimate the pupal age of the *S. peregrina*. Future studies will include more of the influencing factors, such as fluctuating temperatures, to better estimate the pupal age in more realistic forensic investigations.

## Figures and Tables

**Table 1:** Identification of major characteristic peaks in ATR-FTIR spectroscopy.

Baseline Points (cm^−1^)	Wavenumber (cm^−1^)	Infrared Band
1760~1730	1741	Lipid (C = O stretching vibration)
1680~1610	1647	Amide I (C = O stretching)
1580~1510	1540	Amide II (N-H bending coupled to C-N stretching)
1480~1420	1454	C-H bending from CH2 and CH3
1420~1350	1398	C=O vibrations of COO− from free fatty acids, free amino acids, and polypeptides
1330–1277	1313	Amide III
1180~1100	1145	C-O(H) stretching vibration
1100~1000	1040	C-O(H) or C-C vibrations and PO2- symmetric stretching

**Table 2:** The metrics of the OPLS-DA model based on the CHC profiles and ATR-FTIR spectroscopy of different pupae ages (days) of *S. peregrina* at different constant temperatures.

	OPLS-DA				Permutation Test	
	Temperatures	R2X (cum)	R2Y (cum)	Q2 (cum)	R^2^	Q^2^
CHCs	20 °C	0.965	0.946	0.863	0.272	−0.681
	25 °C	0.971	0.972	0.913	0.285	−0.488
	30 °C	0.928	0.982	0.957	0.308	−0.83
ATR-FTIR	20 °C	1	0.97	0.964	0.0971	−0.29
	25 °C	1	0.978	0.974	0.0545	−0.229
	30 °C	1	0.899	0.953	0.0805	−0.275

**Table 3:** The metrics of the PLS regression model based on the CHC profiles and ATR-FTIR spectroscopy of different pupae ages (days) of *S. peregrina* at different constant temperatures.

	PLS				Permutation Test	
	Temperatures	Equation	R2	RMSECV	R2	Q2
CHCs	20 °C	y = x − 1.316 × 10^−7^	0.9618	1.067	0.258	−0.417
	25 °C	y = x − 3.242 × 10^−7^	0.981	0.702	0.259	−0.745
	30 °C	y = x − 2.37 × 10^−7^	0.9809	0.589	0.344	−0.455
ATR-FTIR	20 °C	y = x + 2.923 × 10^−7^	0.927	1.268	0.00486	−0.267
	25 °C	y = x + 1.74 × 10^−8^	0.988	0.364	0.0197	−0.418
	30 °C	y = x + 8.175 × 10^−8^	0.9957	0.163	0.0353	−0.344

## Data Availability

The data and code presented in this study are available on request from the corresponding author.

## Supplementary Materials

The following supporting information can be downloaded at: https://www.mdpi.com/article/10.3390/insects14020143/s1, Table S1: CHCs profile of different pupae age (days) of *S. peregrina* at different constant temperatures, Table S2: The CHC profiles with VIP > 1 of PLS and OPLS-DA model, Table S3: The ATR-FTIR spectroscopy with VIP > 1 of PLS and OPLS-DA model, Figure S1: The OPLS-DA model from the CHC profiles and ATR-FTIR spectroscopy of different pupae age (days) of *S. peregrina* at different constant temperatures (A: 20 °C, B: 25 °C, C: 30 °C) are validated by 50 random permutation tests, whose results indicate the model is acceptable, Figure S2: The PLS regression model from the CHC profiles and ATR-FTIR spectroscopy of different pupae age (days) of *S. peregrina* at different constant temperatures (A: 20 °C, B: 25 °C, C: 30 °C) are validated by 50 random permutation tests, whose results indicate the model is acceptable.
