# Supplementary figures and images for: Pupal Age Estimation of Sarcophaga peregrina (Diptera: Sarcophagidae) at Different Constant Temperatures Utilizing ATR-FTIR Spectroscopy and Cuticular Hydrocarbons
Source: Insects. 2023 Jan 31;14(2):143. doi: 10.3390/insects14020143 (PMC9965786; doi:10.3390/insects14020143)

Figure S1

**CHCs**

(A)

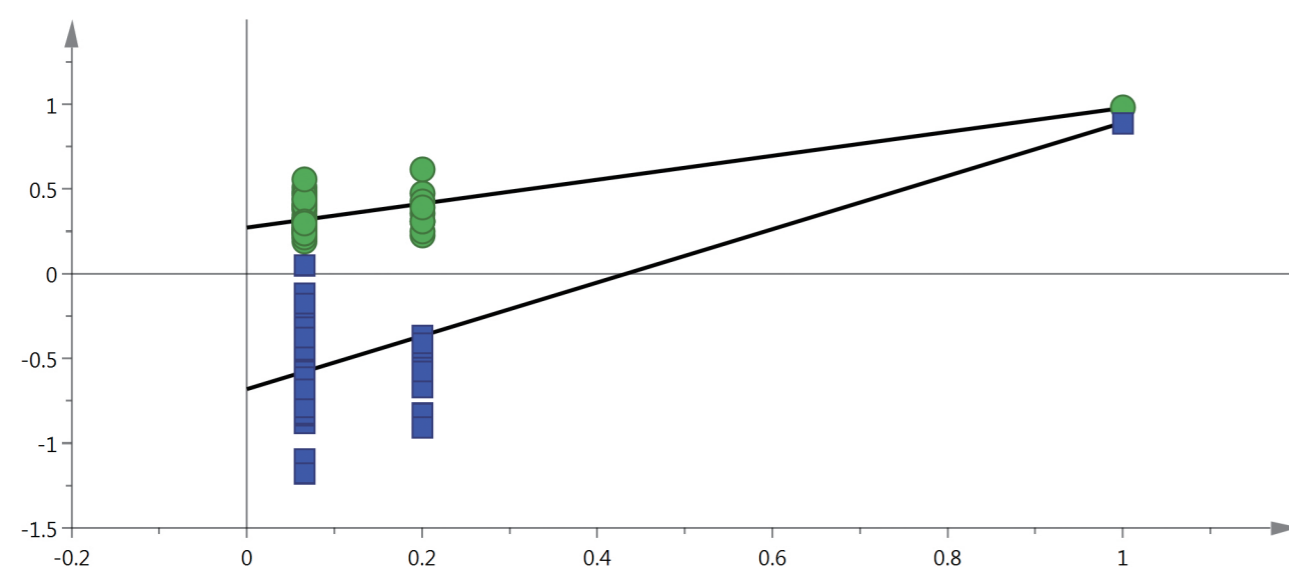

(B)

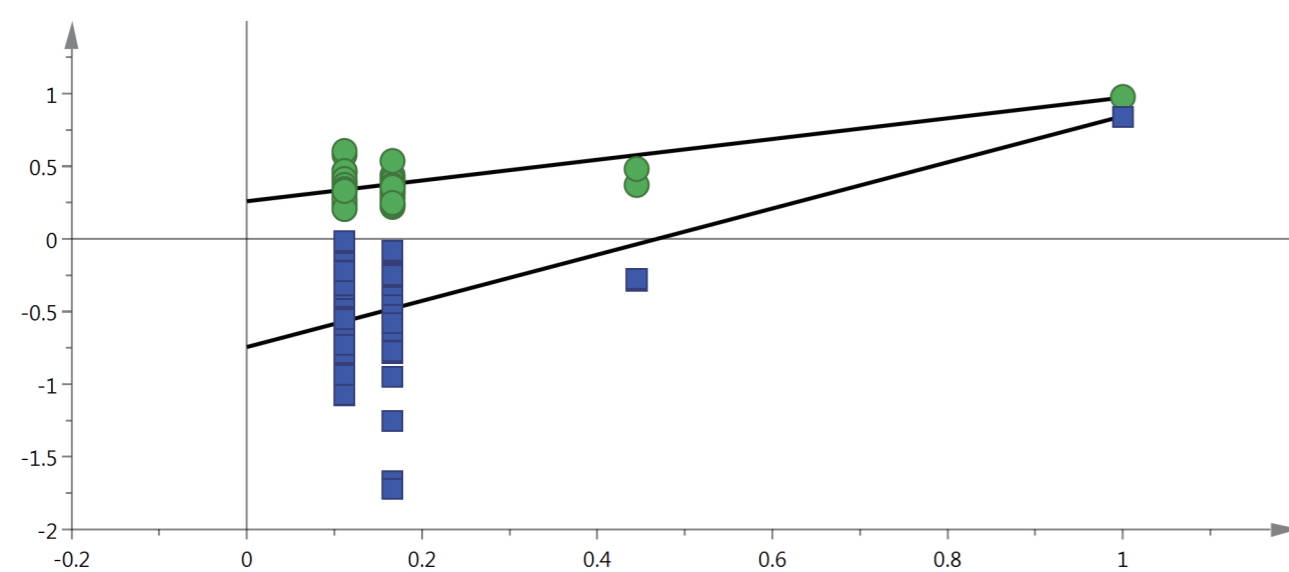

(C)

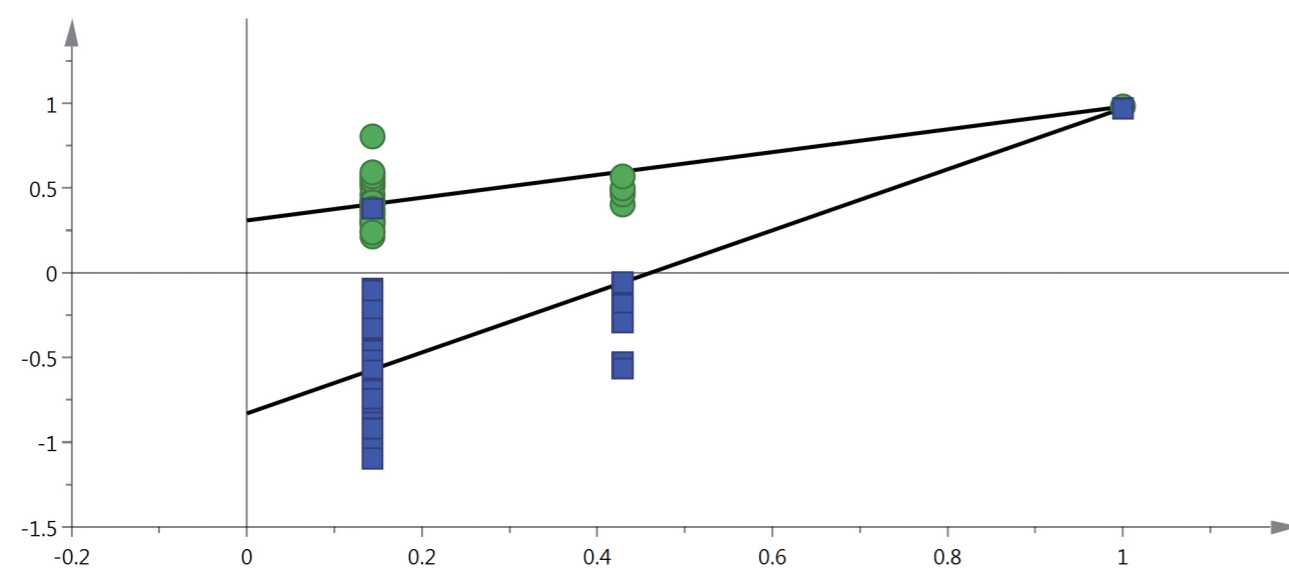

**ATR-FTIR**

(A)

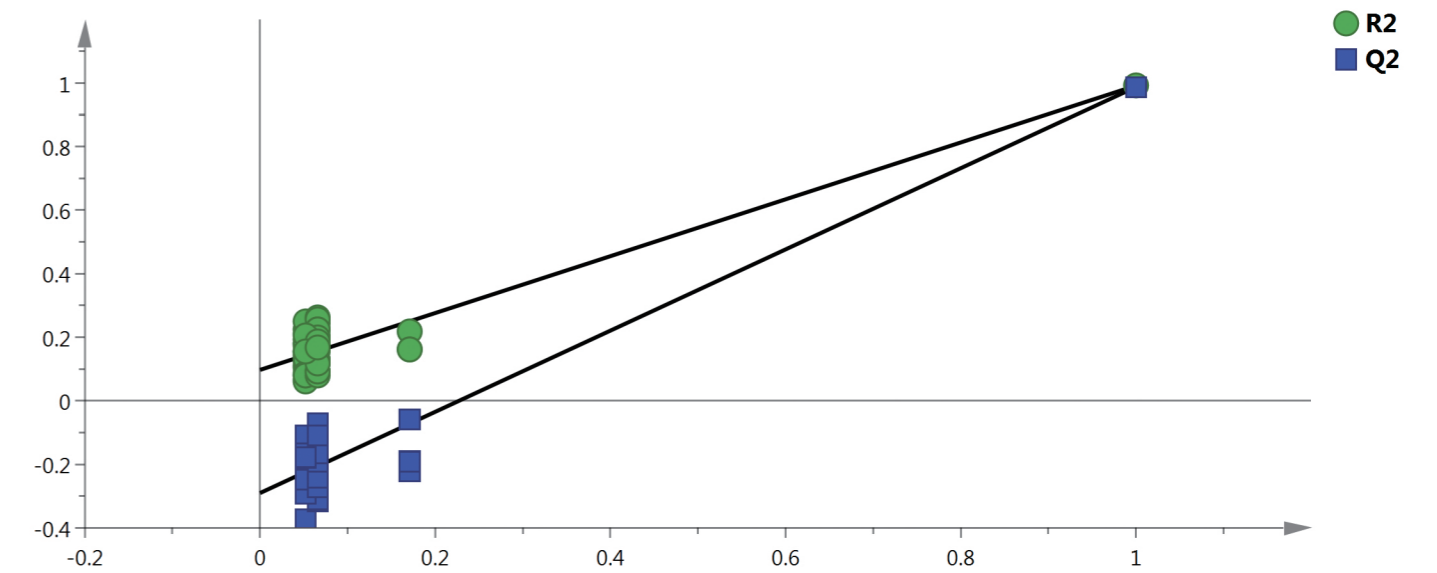

(B)

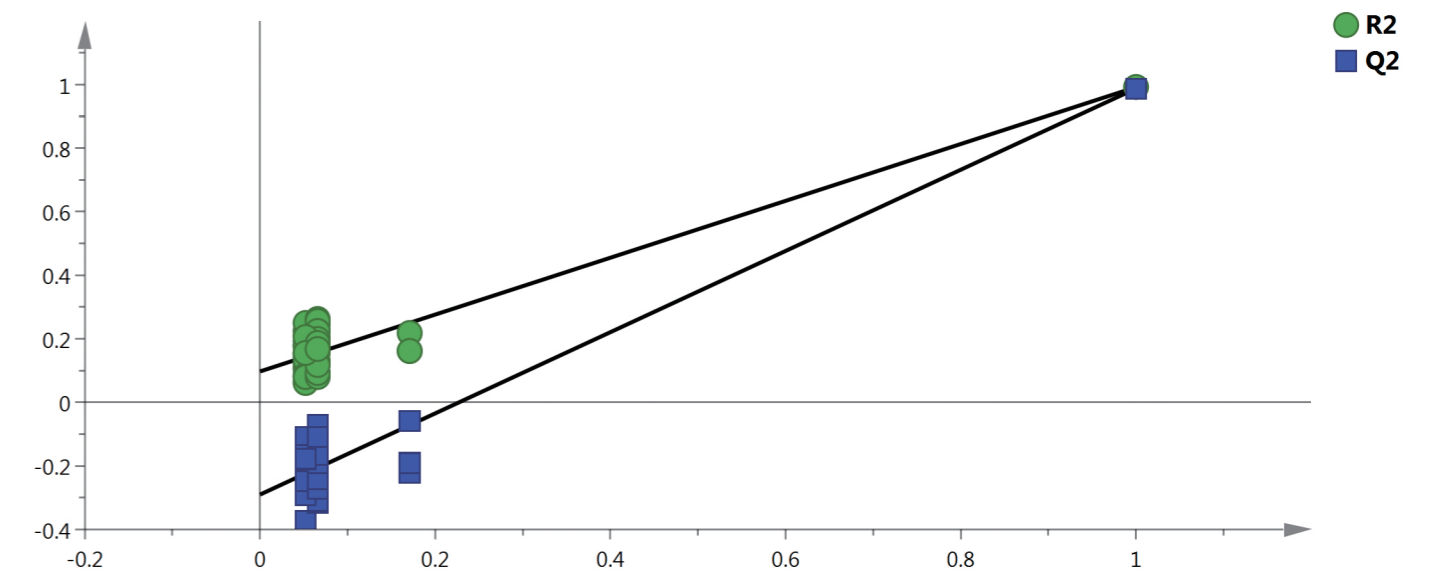

(C)

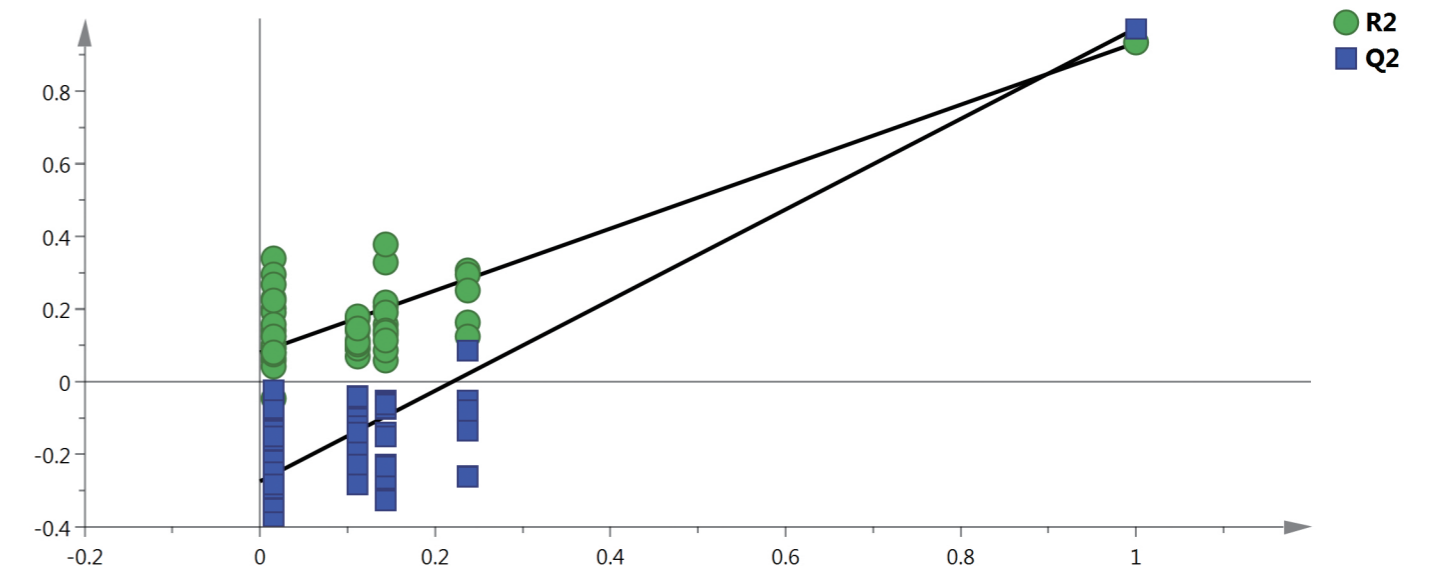

Figure S2

CHCs

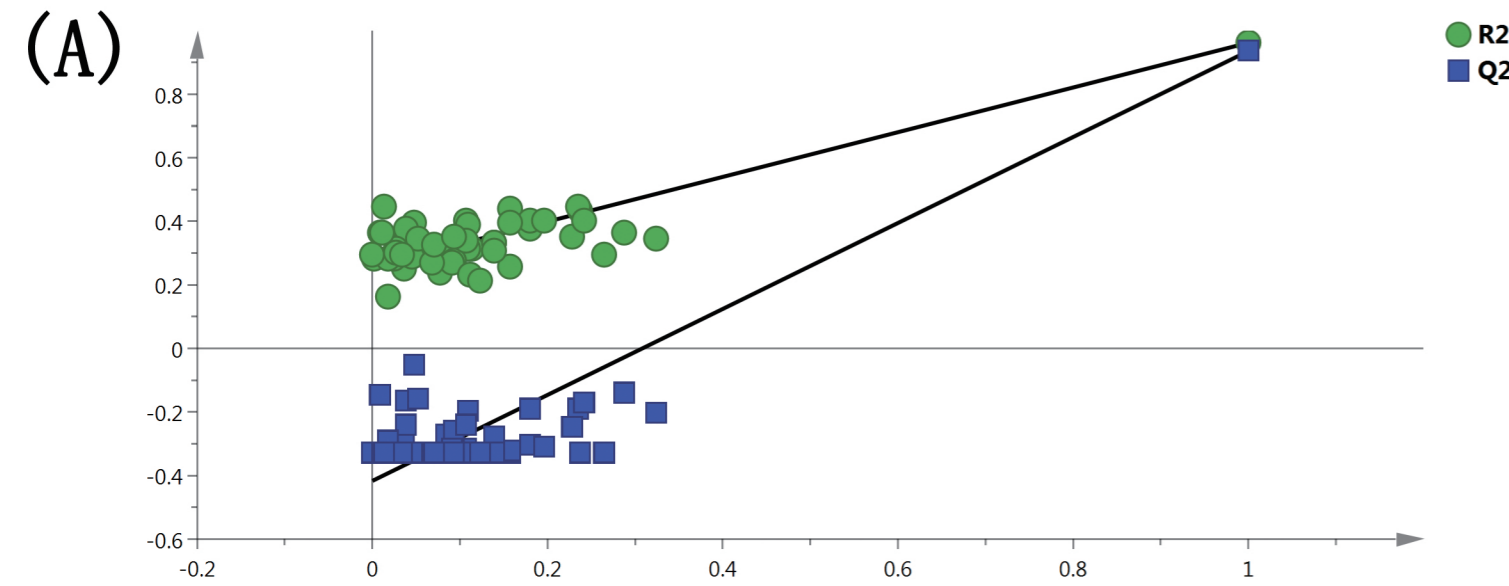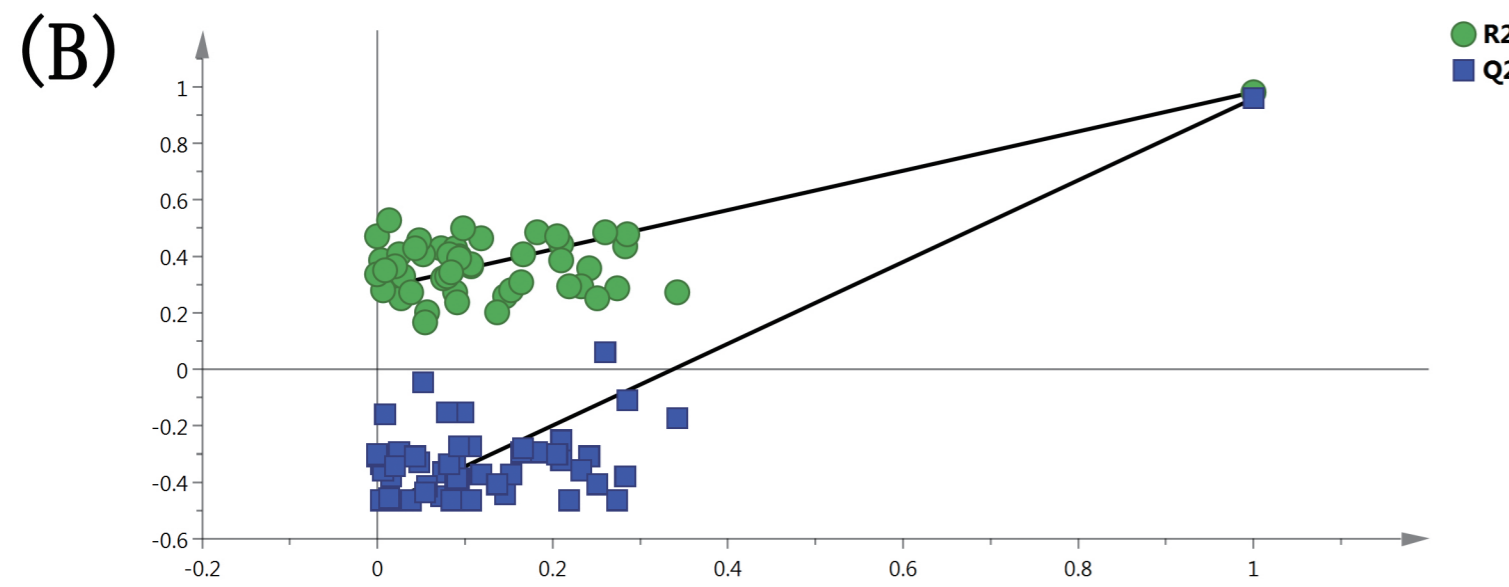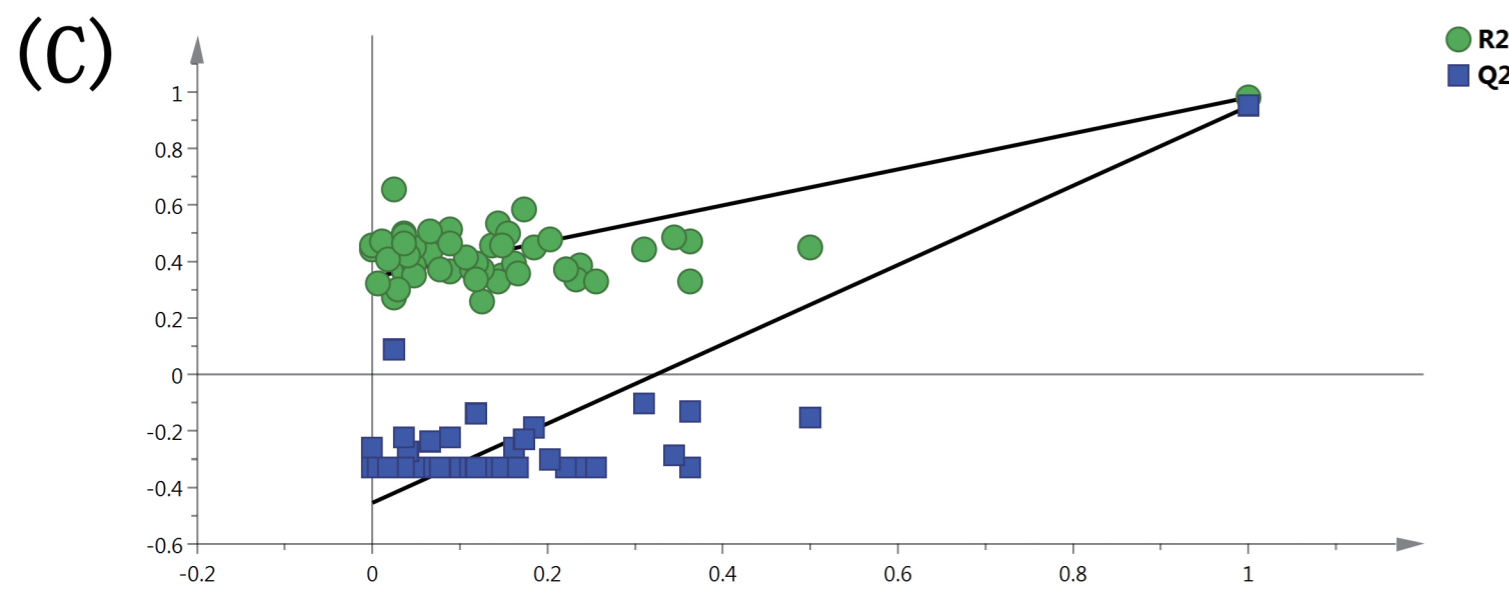

ATR-FTIR

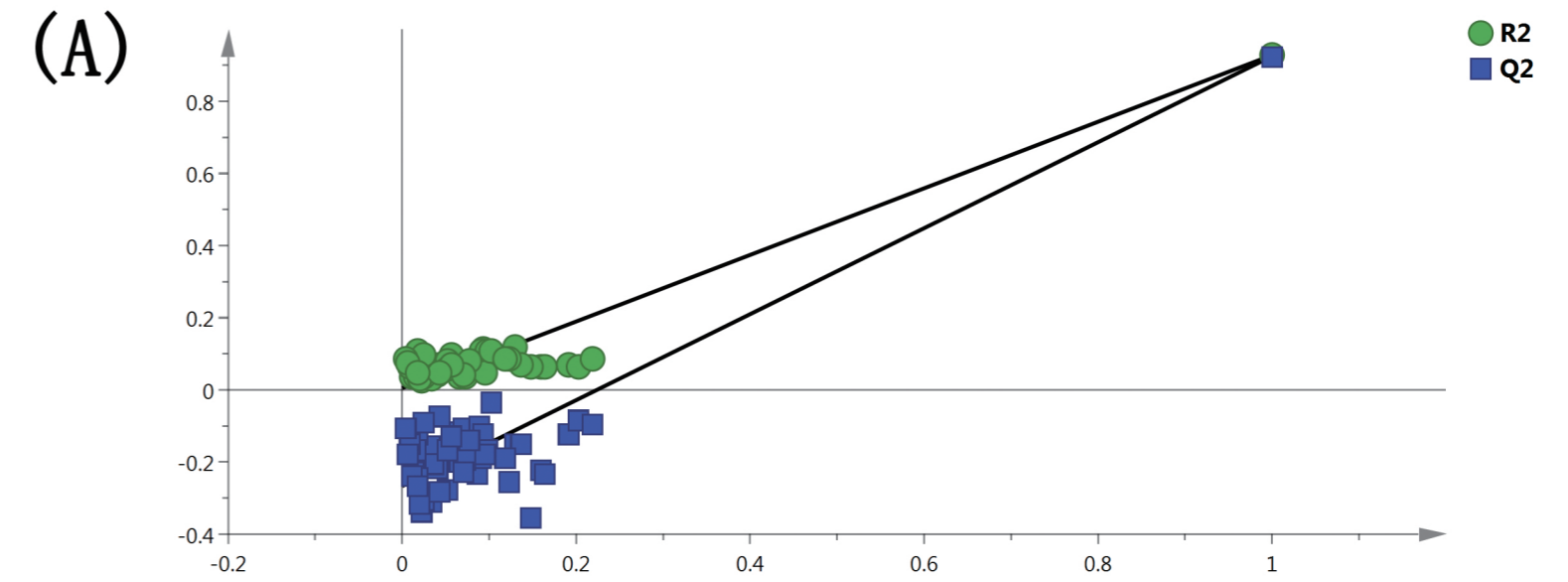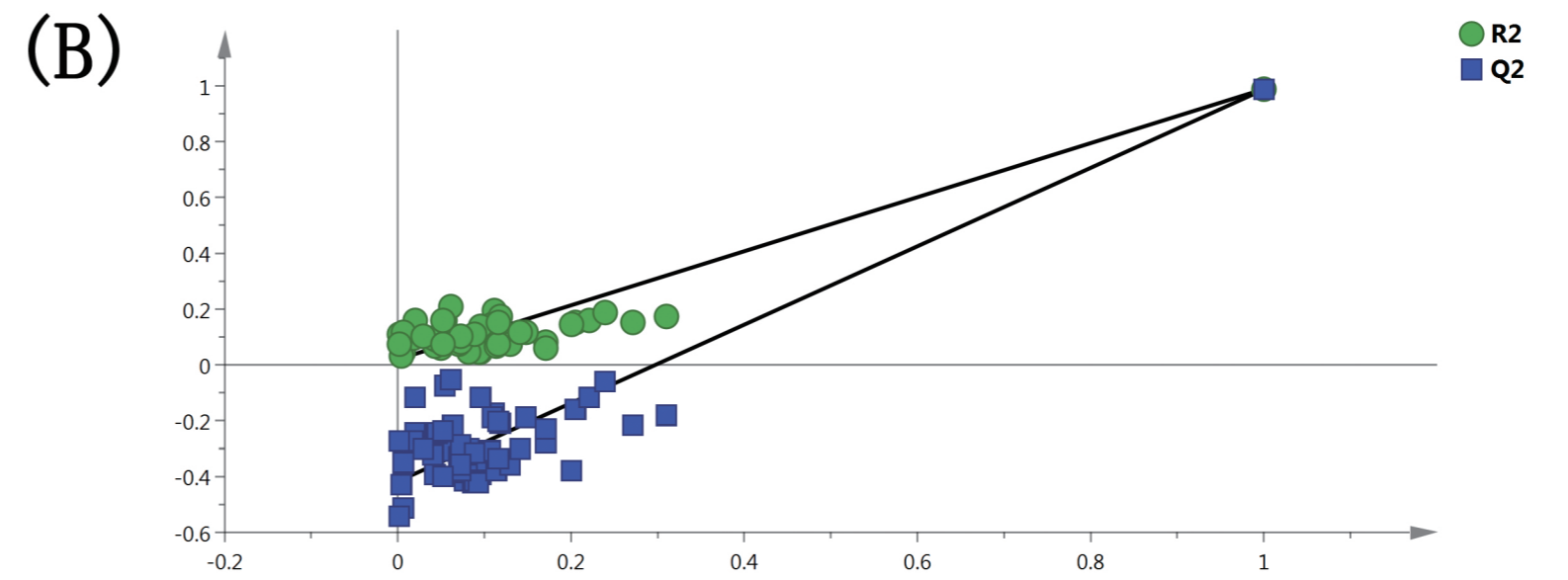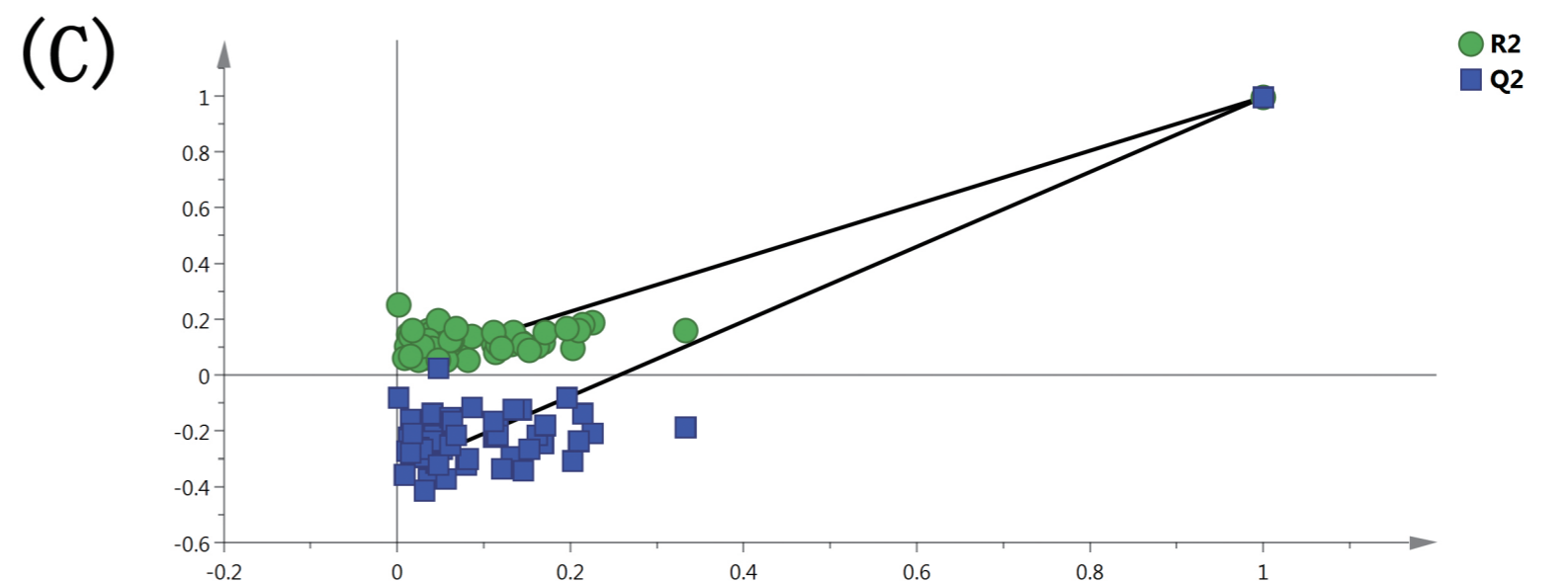

Supplement: Supplementary file 1 [file insects-14-00143-s001.zip › insects-supplementary-Figure.pdf]
